# Non-invasive in vitro NAM for the detection of reversible and irreversible eye damage after chemical exposure for GHS classification purposes (ImAi)

**DOI:** 10.1007/s00204-024-03940-x

**Published:** 2025-01-08

**Authors:** Nicola Knetzger, Norman Ertych, Tanja Burgdorf, Joelle Beranek, Michael Oelgeschläger, Jana Wächter, Annika Horchler, Stefanie Gier, Maike Windbergs, Susann Fayyaz, Fabian A. Grimm, Georg Wiora, Christian Lotz

**Affiliations:** 1 Translational Center Regenerative Therapies (TLC-RT), Fraunhofer Institute for Silicate Research (ISC), Röntgenring 12, 97070 Würzburg, Germany; 2 Department Experimental Toxicology and ZEBET, German Centre for the Protection of Laboratory Animals (Bf3R), German Federal Institute for Risk Assessment (BfR), Berlin, Germany; 3 Institute of Pharmaceutical Technology, Goethe University Frankfurt (GU), Frankfurt Am Main, Germany; 4 Clariant Produkte (Deutschland) GmbH, Frankfurt Am Main, Germany; 5 Courage + Khazaka Electronic GmbH (C+K), Cologne, Germany; 6 Tissue Engineering and Regenerative Medicine (TERM), University Hospital Würzburg, Würzburg, Germany

**Keywords:** Eye irritation, NAM, Non-invasive, Impedance spectroscopy

## Abstract

The potential risk of chemicals to the human eye is assessed by adopted test guidelines (TGs) for regulatory purposes to ensure consumer safety. Over the past decade, the Organization for Economic Co-operation and Development (OECD) has approved new approach methodologies (NAMs) to predict chemical eye damage. However, existing NAMs remain associated with limitations: First, no full replacement of the in vivo Draize eye test due to limited predictability of severe/mild damage was reached. Second, the existing NAMs do not allow reliable differentiation between reversible and irreversible eye damage. Especially the prediction of tissue recovery remains challenging in vitro. Existing in vitro NAMs are based on destructive analysis with no consideration of tissue recovery. In this study, we developed a standalone eye-irritation test method based on non-invasive impedance spectroscopy (ImAi) to discriminate between damaging and irritating chemicals. Tissue effects were analyzed via transepithelial electrical resistance (TEER) measurements of human in vitro epithelial models over 14 days. The TEER was performed using a developed impedance spectrometer. For development of the EIT, a chemical reference list of 329 chemicals was compiled. The applicability of the ImAi-test was exemplified by the discrimination of Cat. 1 vs. Cat. 2 for 23 reference chemicals. Correct classification was achieved for 90.9% of Cat. 1 and 83.3% of Cat. 2 chemicals. Our non-invasive in vitro test overcomes the limitations of Cat. 2 classification of the existing in vitro methods and provides for the first time a non-animal test method that can fully replace the Draize eye test.

**Supplementary Information:**

The online version contains supplementary material available at 10.1007/s00204-024-03940-x.

## Introduction

Traditionally chemical risk assessment relied on in vivo data that continues to be the standard reference for validating new approach methodologies (NAMs). On behalf of the Food and Drug Administration (FDA), toxicologist John Draize and his colleagues published a protocol for acute toxicity testing, the Draize Eye Irritation Test (EIT) (John H. Draize et al. 1944). Serious eye damage and irritation have historically been assessed using laboratory animals such as albino rabbits, as described in the Organization for Economic Co-operation and Development (OECD) Test Guideline (TG) 405, which was adopted in 1981 and most recently revised in 2017 (OECD 2023b). As standalone method, the Draize eye test classifies tissue damages according to their hazard potential into three UN GHS categories: Category 1 (Cat. 1) for irreversible damages, Category 2 (Cat. 2) for reversible irritations, and No Category (No Cat.) for chemicals not harmful to the eye. For a number of reasons, in particular the high level of animal suffering and the subjective scoring scheme with low reproducibility especially for Cat. 2 substances, alternative test strategies have been developed. In addition, current EU regulations clearly emphasize that animal testing should only be used as a last resort for chemical testing (OECD 2019; European Parliament, Council of the European Union 2006; Barroso et al. 2017; Macmillan et al. 2024). However, while NAMs have been continuously developed and improved, they still do not cover the full range of eye-irritation potential across all chemical functionalities. Furthermore, the NAMs used are not able to comprehensively predict all three chemical classes for eye damage in a single test. Unlike the Draize eye test, most of NAMs assess the eye-hazard potential at a single time point immediately following the initial chemical exposure, with no subsequent analysis conducted regarding the reversibility of effects. This results in complex testing strategies of combining the most predictive NAMs, making testing time and cost intensive.

To address time-dependent tissue effects and overcome limitations of existing NAMs, we aim to improve the reliability of the in vitro test with a non-invasive method of impedance spectroscopy for the discrimination of eye irritations — with focus on distinguishing reversible and irreversible tissue damages in human reconstructed cornea-like epithelium (RCE) models.

### New approach methodologies for eye irritation

To fully replace the Draize eye test, the development of NAMs has focused on serious eye damage and eye irritation in context of the OCED guidance document Integrated Approaches on Testing and Assessment (IATA) (OECD 2019). As one module of the IATA, the TG 467 for Defined Approaches (DA) for Serious Eye Damage and Eye Irritation (OECD 2024a) represents accepted test strategies by the combination of existing NAMs. Two DAs are officially accepted for eye-hazard identification: one DA based on TG 492 (evaluating both tissue models separately) and TG 437 and the other DA based on TG 491 and TG 437 (OECD 2024a). Both DAs, however, rely on TG 437, the Bovine Corneal Opacity and Permeability (BCOP) assay which is based on an ex vivo model. Since 2023, the OECD TG 492B allows full in vitro GHS category identification within a single test as first TG (OECD 2024c). However, the correct detection of GHS Cat. 2 liquids are 79.8% and the correct identification of solids is only 55.3% (OECD 2024c). These predictions are based on cell viability measurements of in vitro tissue models based on the reduction of the dye MTT (3-(4,5-dimethylthiazol-2-yl)−2,5-diphenyltetrazolium bromide), which is applied two hours after chemical exposure. The prediction is not generated in a single test; rather, at each defined time point, in vitro models are used once for an MTT readout which results in an increased number of tissue models for analysis. Due to limitations of the currently available NAMs, which do not yet encompass the prediction of hazard potentials for all chemical groups, a general replacement of the in vivo test is not be recommended. However, the classification of irritants (GHS Cat. 2) is based on relative values compared to viable tissue at a single time point with no observation of tissue recovery. Regarding the time-dependent tissue regeneration following exposure to Cat. 2 chemicals, the predictive capability could be enhanced through methodological improvements, both for in vivo and in vitro methods. Using non-invasive methods such as impedance spectroscopy, tissue recovery can be quantified over time for each single tissue model. As part of the test system, tissue models are based on different sources such as human primary corneal or skin epithelial cells, human corneal cell lines or non-human cells. For example, TG 492 includes tissue models based on human primary keratinocytes (EpiOcular™), primary human corneal epithelial cells (LabCyte CORNEA-MODEL24 and MCTT HCE™) and cornea cell line (SkinEthic™) (OECD 2024b). In accordance with previous work, EITs were also performed using human in vitro models based on primary keratinocytes within the impedance-based eye irritation test (ImAi-test) (Lotz et al. 2018; Weissinger et al. 2024). These models have been tested and proven to be predictive of ocular irritation due to their morphologic similarity to the human non-keratinized corneal epithelium.

### Analysis of tissue recovery via transepithelial electrical resistance

Various testing strategies have been developed to assess the hazard potential of chemicals to the human eye. The choice of the analysis method impacts not only the accurate prediction of eye irritations but also time and costs of the test. Established in vitro NAMs are mostly based on short time approaches using destructive tissue analysis: The OECD TG 492 and TG 492B (OECD 2024c) investigate tissue viability 2 h after treatment by a MTT assay. Due to invasive chemical reaction, further cultivation of the tissue models after MTT measurement is not possible. Limited in its ability to directly analyze reversible eye irritation, MTT analysis of one time point does not provide direct information on tissue regeneration compared to in vivo data. This may represent a significant drawback, as chemicals with reversible damage potential cannot be reliably quantified by real-time measurements.

Non-invasive measurements have already been implemented to classify chemical hazard potential to the human eye. Impedance spectroscopy as non-invasive analysis tools has been established in the adopted OCED TG 494 to predict non-harmful chemicals (GHS No Cat.) (OECD 2023f). Takezawa et al*.* employed the human corneal epithelium (HCE) model to investigate the effects of four mild irritants by analyzing the relative change in TEER at 12.5 Hertz (Hz) over time (Takezawa et al. 2011). According to Yamaguchi et al., the Vitrigel®-Eye Irritancy test method has been used to classify 107 chemicals as either irritants or non-irritants (Yamaguchi et al. 2016). Further studies showed predictions based on impedance spectroscopy to assess eye irritations of in vitro tissue models after chemical exposure mainly at 12.5 Hz (Lotz et al. 2018; Weissinger et al. 2024; Chacón et al. 2019, 2022). In addition to TEER at 12.5 Hz, which is commonly used for in vitro approaches (Sayoc-Becerra et al. 2020; Blume et al. 2010), TEER at 1000 Hz has been well established to assess cell proliferation, barrier integrity and membrane functionality over time in several in vitro applications (van der Helm et al. 2019; Profaci et al. 2020; Liu et al. 2023; Neil et al. 2020; Rohde et al. 2022). The non-invasive method can be applied multiple times without damaging the model, making it suitable for monitoring model developments and wound healing studies (Kiesewetter et al. 2019; Liu et al. 2022). Therefore, tissue recovery following chemical exposure can be measured by relative changes of the electrical resistance over time (Yamaguchi et al. 2013; Chacón et al. 2019, 2022). Using the full spectral measurements of impedance spectroscopy, also phase and amplitude data represent promising readout parameters. Implementing the non-invasive method of impedance spectroscopy aimed to develop a test method that accurately detects, potentially reversible, in vitro tissue damage of chemicals over time. Furthermore, the ImAi-test has been developed to differentiate between severe and mild ocular irritation and overcome limitations of current NAMs, thereby providing a more suitable alternative to the Draize eye test.

### Reference substances

As the in vivo test remains the standard for assessing human eye irritation, the chemicals used in the regulatory tests are important for the establishment and validation of alternative test methods. A comprehensive observation and collection of reference chemicals was done by Cosmetics Europe by Barroso et al*. and* based on this Draize data base (DRD) reference substances were carefully selected and used to establish new test methods (Adriaens et al. 2017). For the ImAi-test, a comprehensive set of test substances with different functional groups was selected based on the DRD in vivo references and including proficiency chemicals of relevant in vitro methods (e.g., TG 492 and TG 494 (OECD 2023f, 2024b)). Compared to in vivo reference data, alternative TGs have limitations about substance classes and indicate a high false positive rate for alcohols (OECD 2023d, 2023c), ketones (OECD 2023c) and high false negative rate for surface-active substances (OECD 2023d), solids (OECD 2023d, 2023c) and acids (OECD 2023f). Gases and aerosols are often not used within TGs, since exposure is difficult to control (OECD 2023d, 2023f). For other problematic substances, such as detergents, formulations, or dilutions, there is only limited reference data available. This refers to the in vivo TG 405, where mainly neat substances are tested with only a limited number of dilutions. (OECD 2023b; Barroso et al. 2017). When compiling the list of reference chemicals for the ImAi-test, the key drivers of classification of ocular irritation in vivo were taken into account.

## Material and methods

### Cell origin

The human tissue used in this study was sourced from the University Hospital Würzburg, Germany. Prior to sample collection, informed consent was obtained from the patients and their legal guardians in accordance with the guidelines set forth in the Declaration of Helsinki. The use of biopsies was granted ethical approval by the ethics commission of University Würzburg, under the protocols 182/10 and 280/18sc.

### Tissue-engineered RCE models

RCE models were generated using a well-established protocol (Lotz et al. 2018). Human Epidermal Keratinocytes (HEKs) at passage 3 were cultured until they reached approximately 80% confluency. To detach the cells, they were treated with Accutase® for a duration of 10 min. Subsequently, the cell suspension was centrifuged at 300 g for 5 min, and the resulting pellet was resuspended in E2 medium (EpiLife™ medium (Life Technologies, Carlsbad, USA) supplemented with 1%-human keratinocyte growth supplement (Life Technologies, Carlsbad, USA), 1%-penicillin/streptomycin (P/S) (Sigma-Aldrich, Schnelldorf, Germany), 0.48% 300-mM CaCl_2_ (Sigma-Aldrich, Schnelldorf, Germany)). The RCE models were then cultured in inserts featuring a polycarbonate membrane with 0.4-µm pores and a culture area of approximately 0.59 cm^2^ (BRAND, Wertheim, Germany). For seeding, 3 × 10^5^ cells were placed in each insert using 300 µl of E2 medium. The inserts were incubated under standard culture conditions, maintaining a temperature of 37 °C, humidity at 95%, and a CO_2_ level of 5% for a period of 2 h. Following the initial incubation, 1.4 ml of E2 medium was added to each well surrounding the inserts. After 24 h of submersed culture, the medium within the inserts was replaced with 1.4 ml of E3 medium (EpiLife™ medium (Life Technologies, Carlsbad, USA) supplemented with 1% human keratinocyte growth supplement (Life Technologies, Carlsbad, USA), 1% P/S (Sigma-Aldrich, Schnelldorf, Germany), 0.48% 300-mM CaCl_2_ (Sigma-Aldrich, Schnelldorf, Germany), 73-µg/ml Ascorbyl-2-phosphate (Sigma-Aldrich, Schnelldorf, Germany), 10-ng/ml keratinocyte growth factor (KGF) (Life Technologies, Carlsbad, USA)) to establish an airlift culture. The medium was regularly refreshed every 2–3 days throughout the subsequent cultivation period.

### Impedance spectroscopy

The barrier function of the RCE models was quantified by impedance spectroscopy. Impedance was measured on day 0 before and after the test (day 0 pre, post), on day 7 and 14 after exposure. The models were placed in a 24-well plate and measuring media containing EpiLife™ medium with 0.48% 300-mM CaCl_2_ and 1% P/S was added to the insert and the well. The automated impedance spectrum measurements were performed using the CellSpectrometer CSM 2100 instrument from Courage + Khazaka electronic GmbH (C + K, Cologne, Germany) with titanium nitride (TiN) coated electrodes in a 24-well format (Fig. 3). The 24-well plate was used to place the models. Measuring media was added to both, the well (1.8 ml) and the insert (500 µl). Impedance spectroscopy was conducted by alternating current and low voltage of 120 mV over a frequency range from 1 to 200,000 Hertz (Hz). The CSM 2100 instrument software was developed to analyze the measurements.

### Eye-irritation test

EITs were performed 9 days after setup of RCE models (Lotz et al. 2018). The test procedure was based on the OECD TG 492 (OECD 2024b). Prior to chemical exposure, tissue models were moisturized with 500-µl E1 medium supplemented with 0.48% 300-mM CaCl_2_ for 30 min. After pre-wet, E1 medium was removed and 50 µl of the respective test substance was applied on the tissue surface and incubated for 12 min at room temperature. The substance was removed by three washing steps, in which the models were immersed in beakers filled with 100 ml of PBS. Next, the RCE models were transferred to a 12-well plate filled with 4.5-ml measurement media (EpiLife™ supplemented with 0.48% 300-mM CaCl_2_) and incubated for 12 min at room temperature. For subsequent culturing, the models were transferred to a 24-well plate with 1.4-ml E3 culture media er well. Deionized water was used as the negative control and benzalkonium chloride (10%) as the positive control. The tissue-membrane barrier functionality was identified by *TEER*_*1000Hz*_ on day 0 before normalized to TEER after the application and on days 7 and 14. The test substances were tested in triplicates in three independent runs (*N* = 3; *n* = 3).

### Prediction model

**Fig. 1:**
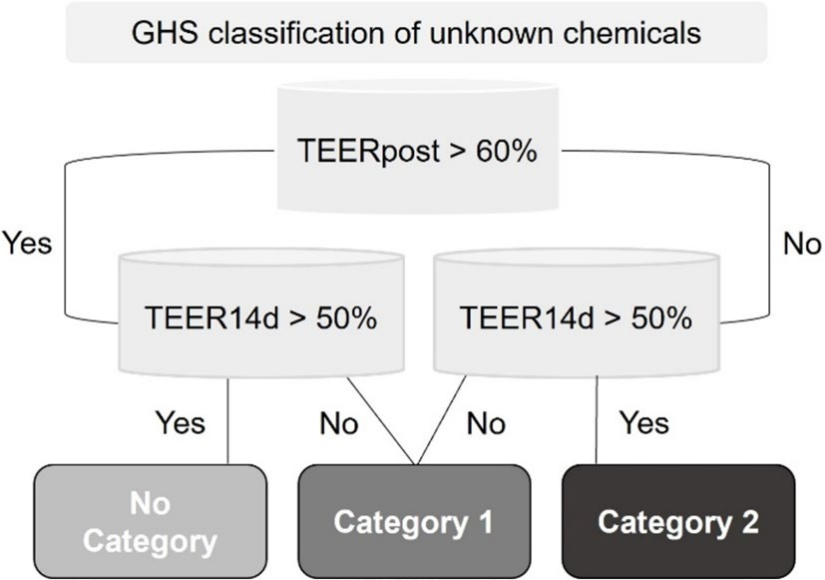
Prediction model to identify the hazard potential of unknown chemicals adapted from Lotz et al. (Lotz et al. 2018) with *TEER*_*1000Hz*_-based prediction of chemical damage potential after treatment (TEERpost) until day 14 (TEER14d) after exposure

Chemical hazard potential can be predicted by assessing the membrane barrier functionality of the treated tissue models using impedance spectroscopy. Based on the UN GHS categories of chemicals, the ImAi-test predicts the tissue integrity by *TEER*_*1000Hz*_ values at several time points. All TEER measurements taken after the application, including TEERpost, TEER7d and TEER14d, are normalized to the TEERpre of each model. The prediction model is based on the mean of the triplicates treated with the same chemical. Each model of a condition is normalized to its corresponding TEERpre. The ImAi-GHS prediction is based on the existing prediction model (Lotz et al. 2018) with extension of time until day 14 (Fig. 1). To predict a Cat. 1 chemical, two options are possible: if the reduction of TEER to less than 60% after application (TEERpost) and TEER less than 50% on day 14 (TEER14d) in relative to TEER before test (TEERpre); or if TEERpost values are over 60% but under 50% on day 14, chemicals are also classified as Cat. 1. In case of Cat. 2 chemicals, TEERpost values are lower than 60% after treatment and higher than 50% on day 14. If the mean TEER exceeds both thresholds on the respective days, chemicals are classified into GHS No. Cat. chemicals.

### Quality control of the tissue models by impedance spectroscopy

To ensure the reproducibility of the GHS classification, the quality of the tissue models was controlled on day 0 before the test. Only tissue models that passed the quality control were used for the EIT. Untreated tissue models represent intact tissue integrity and membrane barrier functionality. For quality control, untreated tissue models were measured and *TEER*_*1000Hz*_ values were analyzed. TEER values (Ohm*cm^2^) were calculated by multiplying the raw TEER value by the surface area of the insert used (BRAND, Wertheim, Germany; surface area = 0.59 cm^2^). The tissue quality criteria are based on: TEER values at the lower limit must be ≥ 600 Ohm*cm^2^ and TEER values at the upper limit must be ≤ 3000 Ohm*cm^2^ (Table 1). Models that fail the quality range could lead to misclassification of applied chemicals and should not be used for the EIT. A total of three biologic donors were used and quality control was performed on each donor prior to testing. The tissue variability of triplicates should be below 18% according to OECD TG 482B (OECD 2024c). The TEER quality control ensures the reproducibility of membrane barrier properties across different human donors enabling correct GHS classification. For negative controls, TEER values were between the limits of ≥ 600 ≤ 3000 Ohm*cm^2^ throughout the EIT. Positive controls should have TEER values consistently lower than 300 Ohm*cm^2^ after chemical treatment. Tissue models and culture medium were free of contamination during the test performances.

**Table 1:** Quality control of the tissue models, negative, and positive control based on measured *TEER*_*1000Hz*_ values. Quality control was performed on day 0 before the test (pre)

Condition	Time (day)	Quality criteria: *TEER*_*1000HZ*_ (Ohm) * Insert area (cm^2^)
Tissue models	0 pre	≥ 600 Ohm*cm^2^ and ≤ 3000 Ohm*cm^2^
Negative control	0 pre 0 post 7	≥ 600 Ohm*cm^2^ and ≤ 3000 Ohm*cm^2^ ʺ n.a.ʺ ʺ n.a.ʺ
Positive control	0 pre 0 post 7	≥ 600 Ohm*cm^2^ and ≤ 3000 Ohm*cm^2^ ≤ 300 Ohm*cm^2^ ʺ n.a.ʺ

### Statistics

The data were analyzed using GraphPad Prism 6 (GraphPad Software, San Diego, USA). Statistical analysis comprised a two-way ANOVA within a Tukey’s multiple comparisons test using alpha ≤ 0.05 for significance with *n* = 6 per group and ten groups in total. Significance was compared between TEERpost vs. TEERpre; TEERpost vs. TEER14d measurements.

## Results

To improve the identification of chemical-induced corneal injuries, we developed a test method to analyze tissue damage over time using impedance spectroscopy. The impedance-based eye irritation test (ImAi-test) has proven successful discrimination between irreversible and reversible induced tissue damages caused by neat liquids and dilutions via TEER.

During ImAi-test development the following steps were reached: (1) The establishment of a list of 329 reference chemicals including a selection of a training set (40–70 chemicals) and a validation set (30 chemicals, marked with*) (supplementary material). The test development was made on a collection of suitable reference chemicals from the DRD of Barosso et al. (Barroso et al. 2017). (2) The development of an impedance measurement device to analyze human tissue models generated in conventional inserts in a 24 well format, allowing a wide range of applications. (3) 23 liquids of the training set were selected to demonstrate the ImAi-test method. Hereby, based on human 3D RCE models, the ImAi-test was able to discriminate liquids causing irreversible (GHS Cat. 1) and reversible effects (GHS Cat. 2) by TEER over time.

### Establishment of reference list for eye-irritation testing

**Fig. 2:**
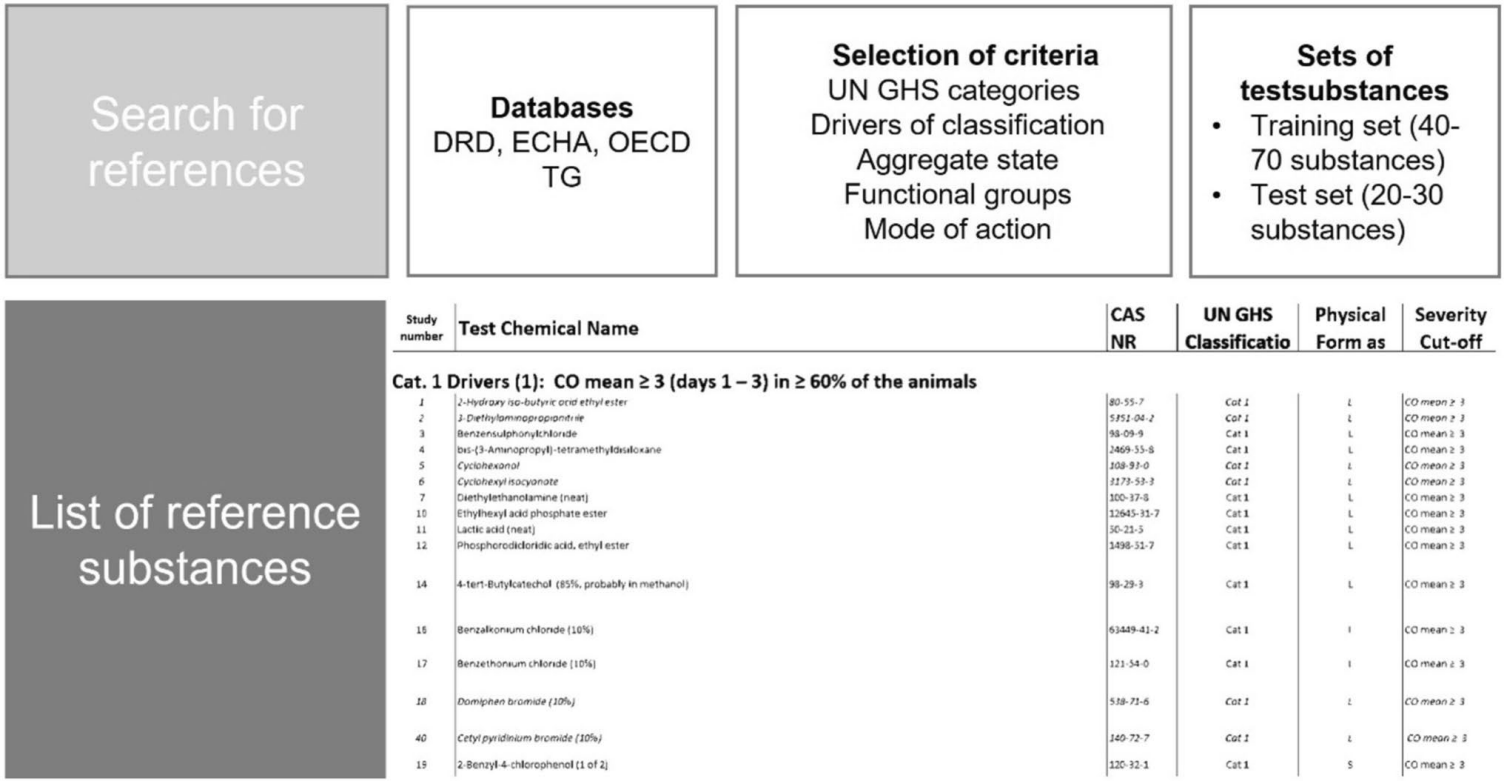
Graphical abstract outlining the procedure for creating the list of test substances to evaluate eye irritations

An extensive list of reference substances was compiled to develop the EIT protocol. Databases such as the DRD Draize eye test Reference Database published by Cosmetics Europe (Barroso et al. 2017), ECHA and OECD TGs were screened to identify suitable reference chemicals (Fig. 2).

Chemicals were identified as suitable if reliable in vivo data were available. To cover a wide range of chemical properties and substance classes, the selection was mainly based on five criteria: (1) the selected substances cover UN GHS categories, (2) the representation of important in vivo drivers of classification based on effects on corneal opacity (CO) and conjunctival redness (CR), (3) the aggregate state of the chemicals including liquids, semi-solids and solids, (4) a wide range of functional groups and 5) different modes of action of the chemicals on the human cornea.

Solid and liquid chemicals were selected of each GHS category (Cat.) such as Cat.1 (26 liquids/29 solids), Cat. 2 (35 liquids/19 solids), No Cat. (122 liquids/72 solids). According to 2), the following drivers of classification for Cat. 1 by order of importance were taken into account: (1) CO mean ≥ 3 (days 1–3) in ≥ 60% of the animals; (2) CO persistence on day 21 in ≥ 60% of the animals (with CO mean < 3); (3) CO = 4 in ≥ 60% of the animals in the absence of persistence and with CO mean < 3 (or if unknown). For Cat. 2, by order of importance: (4) CO mean ≥ 1; (5) CR mean ≥ 2 (with CO mean < 1). Subgroups for chemicals that do not require classification (No Cat.): (6) CO > 0 (minor effects on CO observed); (7) CO = 0 (clear negative results); (8) CO = 0 ** and CO > 0 ** (only a few chemicals should be included). The test substance list is sorted according to the drivers of classification (1)—(8) (supplementary material, Table S1). To cover a broad spectrum of chemicals: 4) in principle, no chemical functional groups were excluded; alcohol, ether, carboxylic acid (ester) are most frequently mentioned; chemical functional groups such as alcohols or ketones have also been included as references, however, it is recognized that an increased false negative rate is reported in some OECD TGs (OECD 2023c, 2023d). Dye and MTT-reducing test substances have also been included in the reference list as, in contrast to MTT-based endpoint measurement, these test substances do not interfere with barrier measurement (supplementary material, Table S1, Reference list, marked with^C^). Furthermore, chemicals inducing cell lysis (surfactants, organic solvents, ketones, alcohols, volatile liquids, ethers, polyether’s, esters, aromatic amines), coagulation (acids, cationic surfactants, organic solvents) and ester hydrolyses (alkalis) were included in the reference list. Reactions with macromolecules (peroxides), mustards, alkyl halides, epoxides, bleaches (oxidizers) were not included due to lack of high-quality in vivo data. The developed reference list is based on 329 chemicals of all three UN GHS categories (supplementary material, Table S1) with 26 liquids and 29 solids of category 1 (Cat. 1), 35 liquids and 19 solids of category 2 (Cat. 2) as well as 122 liquids and 72 solids of no category (No Cat.). Due to the high number of suitable No Cat. chemicals of the DRD, the proportion of No Cat. chemicals also predominate in the ImAi-test reference list. Not many should be included, however, to complete the reference list, 21 liquids and five solids with CO = 0 ** and CO > 0 ** were also listed (supplementary material, Table S1). To define a test-set to validate the ImAi-test, 30 chemicals were selected from the reference list (supplementary material, Table S1, marked with*). The validation test set is based on 10 chemicals of each GHS category, including 5 liquids and 5 solids. The selection is partially based on proficiency chemicals of existing TG (OECD 2024c, 2023c, 2024b, 2023d, 2023f, 2023e). Based on the training set, the ImAi-method was developed to further optimize the prediction model to discriminate all Cat. 1 and Cat. 2 chemicals. In addition to published reference lists for eye-irritation testing (Adriaens et al. 2017; Barroso et al. 2017), the established ImAi-reference list could also support the NAM-development for ocular hazard assessment.

### Development of an impedance spectroscopy measuring device

**Fig. 3:**
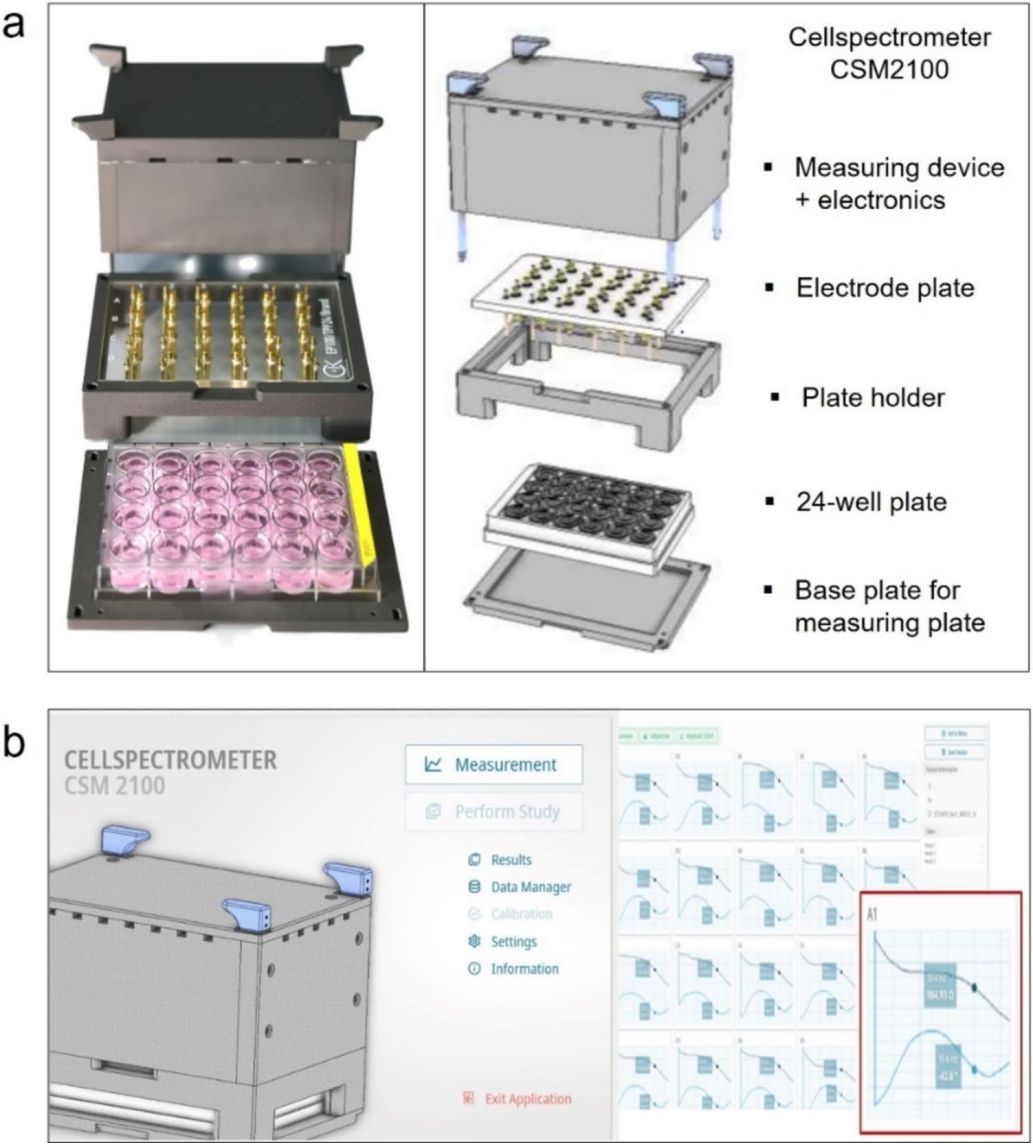
Impedance spectroscopy measuring device and software to analyze tissue properties over time. **a** overview of the construction of the CellSpectrometer CSM 2100. **b** software to analyze and export impedance spectroscopic measurements

**Fig. 4:**
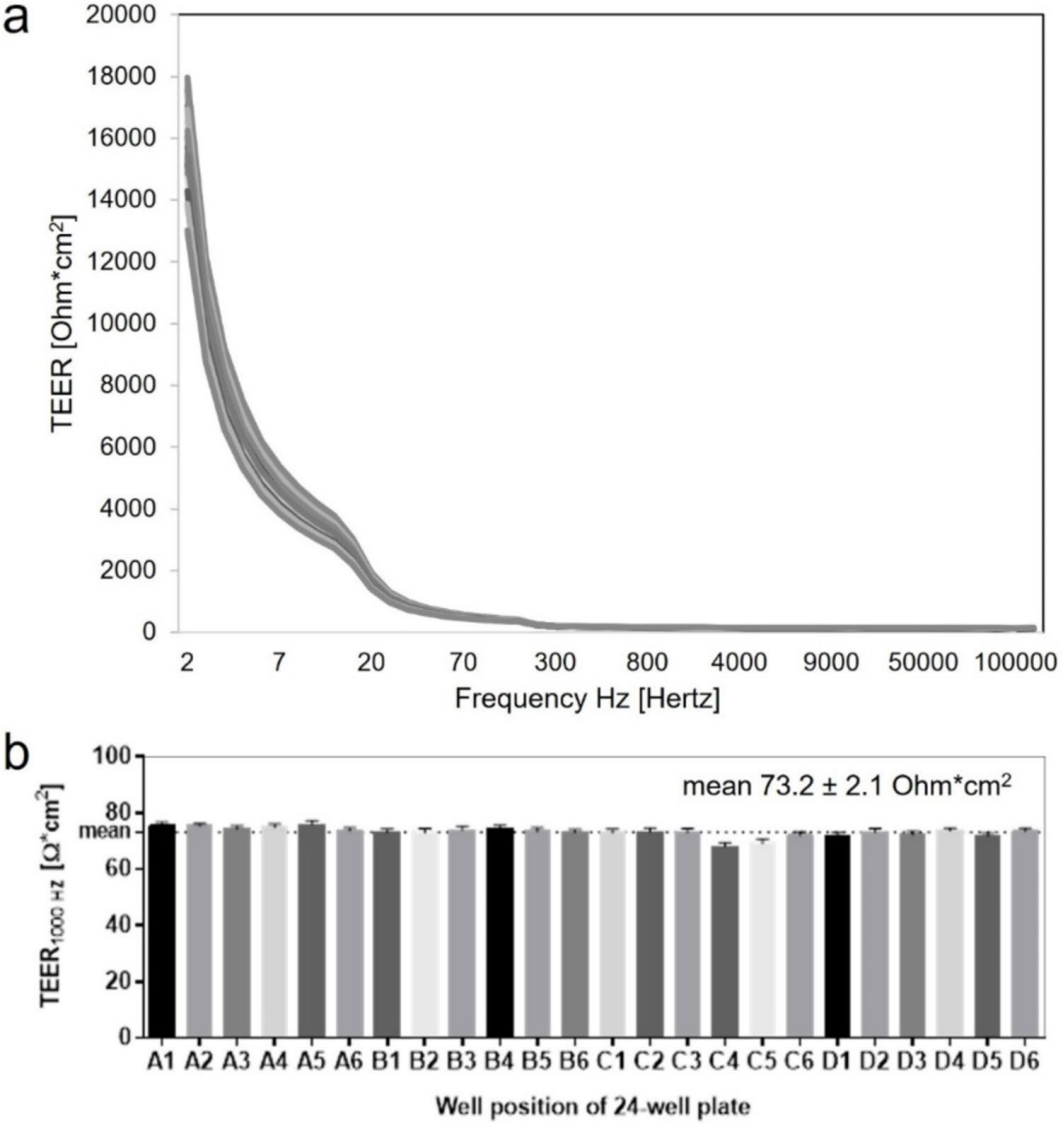
Reproducible impedance spectroscopic measurements of the TIN electrodes. 24 inserts filled with 500-µl impedance measuring media were placed in a 24-well plate containing 1.8-ml measurement media per well. Measurements were performed without cells to ensure comparable and robust resistance of electrodes and media. **a** impedance spectroscopic measurements from 1 to 2 00.000 Hertz of 24 inserts at each well position. **b** *TEER*_*1000Hz*_ values of a 24-well plate showing a mean of 73.2 ± 2.1 Ohm*cm^2^ of *n* = 10 measurements

As part of the ImAi-test development, an impedance spectroscopy measurement device was specifically designed and adapted to the used in vitro models. The CSM 2100 impedance spectrometer device is capable of non-invasively measuring 24 in vitro models in parallel within a standard 24-well culture plate (Fig. 3). The CSM 2100 spectrometer consists of a main body containing the electronic and provides the connection to the electrodes (Fig. 3a). The electrodes are coated by TiN, which allow sensitive full spectrum measurements of tissue integrity and barrier properties also in low frequencies (Fig. 4a) (Schmitz et al. 2018). The TiN-coated electrodes, are mounted in a glass plate which is arranged by the plate holder. The impedance spectrum of a single tissue model is measured using two electrodes, placed inside of the insert and outside of the insert. With the fixed position of the electrodes, the inserts are always placed in the same order, allowing non-contact measurements of the tissue models in the same position. The measurement setups and experimental study designs are defined in the CSM 2100 software (Fig. 3b). To provide information on the tissue barrier properties, measurements can be taken at single frequencies of 12.5 and 1000 Hertz (Hz) or in full spectrum mode ranging from 1 to 200,000 Hz. Blank values including only medium, and inserts showed reproducible values within all 24 electrodes 73.2 ± 2.1 Ohm*cm^2^ (Fig. 4b). Raw measurements were saved into a database and exported as excel-format (.xlsx). Raw data includes data tables and graphs for further evaluation.

### Discrimination between category 1- and category 2-induced tissue damages following exposure to liquid substances

Tissue damages from both category 1 (Cat. 1) and category 2 (Cat. 2) liquids were assessed by their membrane barrier functionality. Therefore, the tissue integrity of the RCE models were measured directly before the treatment (TEERpre), 120 min after post incubation (TEERpost) and in the following on day 7 (TEER7d) and 14 (TEER14d). The EIT was carried out with 23 liquids of Cat. 1 and 2 of the training set (Table 2). Focusing on reversible damage, 12 Cat. 2 (10 Cat. 2A and 3 Cat. 2B liquids) and 11 Cat. 1 liquids were selected, covering the main drivers of classification: CO mean ≥ 3, CO mean < 3, CO = 4, CO mean ≥ 1, CO mean < 1 (Barroso et al. 2017).

**Table 2:** Category 1 and 2 liquids of the training set tested in the ImAi-test. Chemicals are listed based on the DRD. According to the Globally Harmonized System of Classification and Labelling of Chemicals (GHS), GHS 1 indicates irreversible tissue effects or severe damage to the eye. GHS 2A/2B causes eye irritation and fully reversible effects within 21/7 days

DRD Nr	Test substance	Cas Nr	GHS	Drivers of classification
3	Benzensulphonylchloride	98-09-9	1	a; CO pers D21
10	Ethylhexyl acid phosphate ester	12,645-31-7	1	a
14	4-Tert-Butylcatechol (85%, probably in methanol)	98-29-3	1	a; CO pers D21; Conj pers D21
17	Benzethonium chloride (10%)	121-54-0	1	a; CO pers D21 (at least)
34	Hydroxyethyl acrylate	818-61-1	1	CO pers D21 (at least)
68	Cetylpyridinium chloride (10%)	6004-24-6	1	b
69	Cetyltrimethyl ammonium bromide (CTAB) (10%) (1 of 2)	57-09-0	1	b
71	Di(2-ethylhexyl)sodium sulphosuccinate (10%)	577-11-7	1	b
77	Stearyltrimethylammonium chloride (10%)	112-03-8	1	b
129	N-Octylamine	111-86-4	1	c; CO pers unknown
**	1-Hexadecanaminium, N, N, N-trimethyl-, chloride (25%)	112-02-7	1	CO pers D21
167	2-Ethyl-1-hexanol	104-76-7	2A	d
170	Cyclopentanol	96-41-3	2A	d
173	Gamma-Butyrolactone (1 of 2)	96-48-0	2A	d
176	Methyl acetate (1 of 2)	79-20-9	2A	d
180	N-Hexanol	111-27-3	2A	d; CO pers unknown
183	Propasol Solvent P	1569-01-3	2A	d
187	Lauryl sulphobetaine (10%)	14,933-08-5	2A	d
202	Furfural	98-01-1	2A	e; CO pers D7; Conj pers D7
204	Methyl cyanoacetate	105-34-0	2A	e; CO pers unknown; Conj pers D7
209	N-Lauroyl sarcosine Na salt (10%)	137-16-6^*^	2A	d; CO pers unknown
218	2-Methyl-1-pentanol	105-30-6	2B	d; CO pers unknown
219	3-Chloropropionitrile	542-76-7	2B	d; CO pers unknown

^*^7631-98-3 corresponds to N-Dodecyl sarcosine Na salt or Sodium lauryl sarcosine (Gautheron et al. 1994)

^**^Test substance is not included in DRD but referred to the DA of surfactans (DASF) (Alépée et al. 2023)

a) Cat 1 Drivers (1): CO mean ≥ 3 (days 1–3) in ≥ 60% of the animals

b) Cat 1 Drivers (2): CO persistence on day 21 (D21) in ≥ 60% of the animals (with CO mean < 3)

c) Cat. 1 Drivers (3): CO = 4 in ≥ 60% of the animals in the absence of persistence and with CO mean < 3 (or if unknown)

d) Cat. 2 Drivers (4): CO mean ≥ 1; Conj mean ≥ 2; CO pers D7 (or marked if unknown)

e) Cat. 2 Drivers: (5) CR mean ≥ 2 (with CO mean < 1); Conj mean ≥ 2

**Fig. 5:**
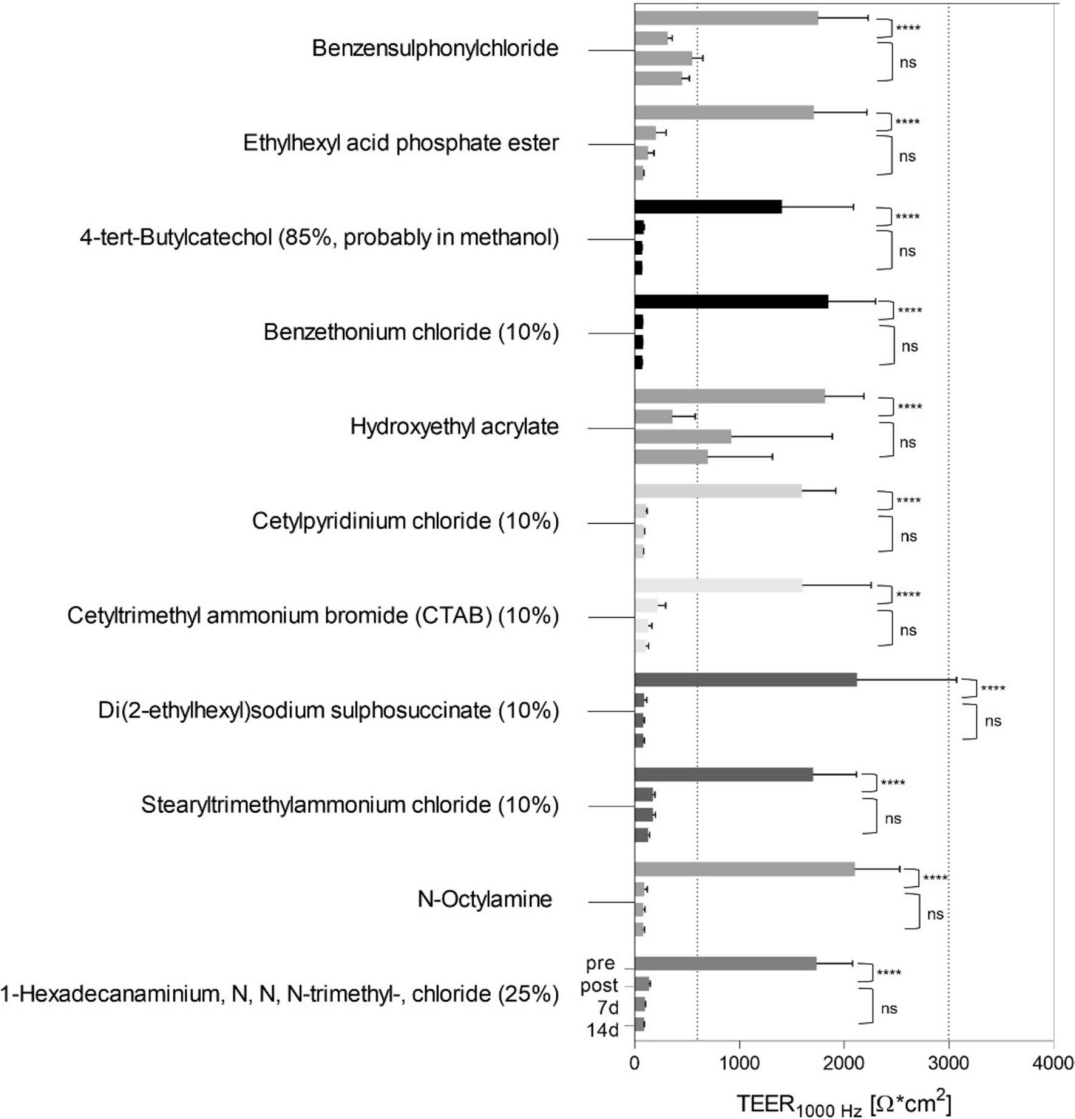
Raw *TEER*_*1000Hz*_ (Ohm*cm^2^) measurements of treated in vitro RCE models indicated strong tissue damages after exposure to 11 category 1 liquids. TEER measurements were taken before (pre), after (post) the test on day 0 and on days 7 and 14 after the test (7d, 14d). Statistical comparison was made of pre vs. post and of post vs. 14d values. Measurements were performed using *N* = 3; *n* = 3. Statistics are based on Tukey’s multiple comparisons test using alpha ≤ 0.05 for significance

**Fig. 6:**
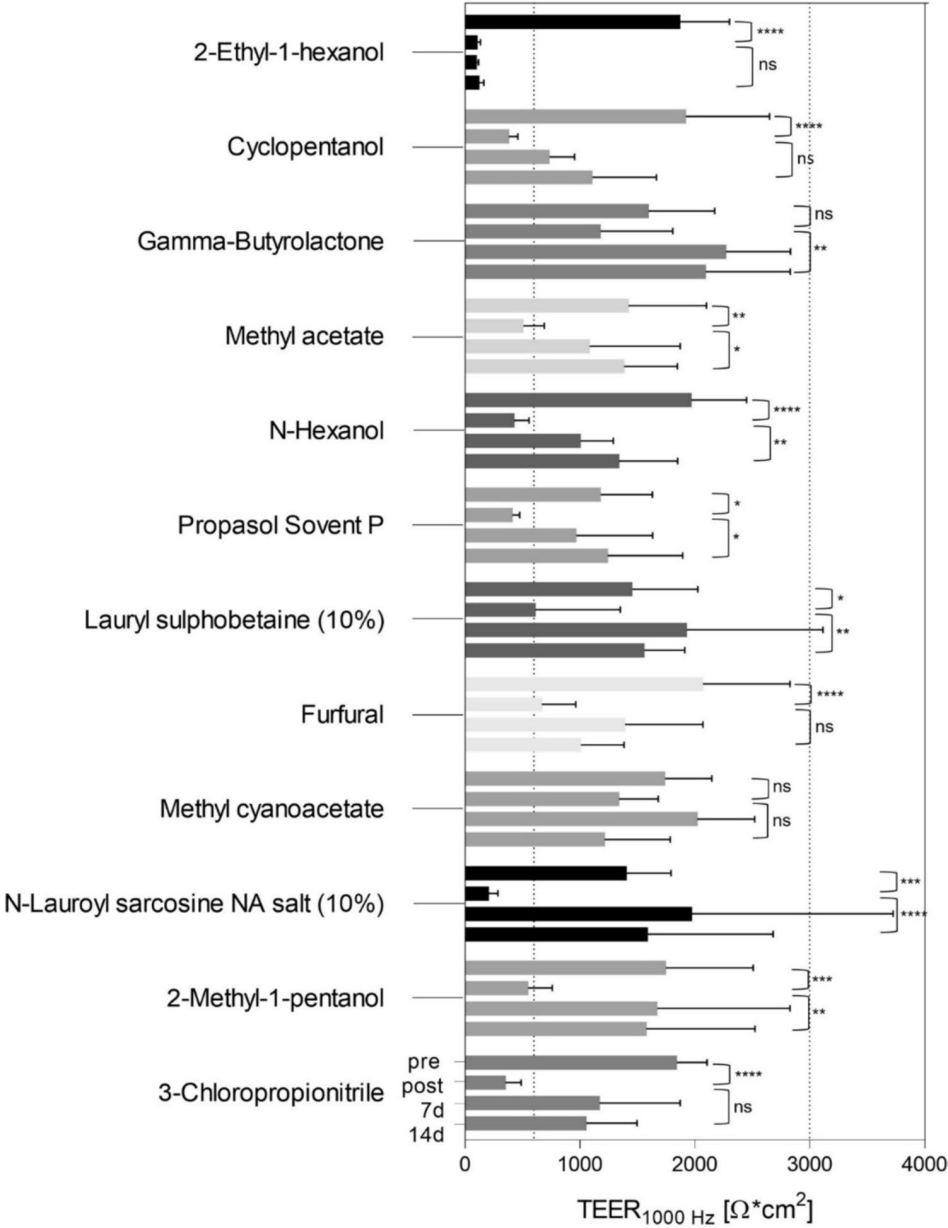
Tissue damage and recovery were observed in treated RCE models exposed to 12 category 2 liquids indicated by raw *TEER*_*1000Hz*_ (Ohm*cm^2^) values. TEER measurements were taken on day 0 before (pre) and after (post) the test and on days 7 and 14 after the test (7d, 14d). Values of pre vs. post and post vs. 14d were statistically compared. EIT was performed using technical triplicates of three different biologic donors. Results are based on Tukey’s multiple comparisons test (alpha ≤ 0.05 for significance)

Results of TEER-measurements of Cat. 1 and Cat. 2 liquids were represented as mean of raw TEER-values (Ohm*cm^2^, Figs. 5, 6) and as mean of normalized TEER-values (%) (Table 3).

**Table 3:** The hazard potential of category 1 and 2 liquids was assessed using normalized TEER values at day 0 after (0d post) and 14 days after the test (14d post). Liquids were classified into ImAi-GHS categories using the prediction model of Lotz et al. (Lotz et al. 2018) and were compared to the UN GHS classification (OECD 2023b)

DRD Nr	Test substance	Cas Nr	UN- GHS	TEER 0d post %	TEER 14d post %	ImAi- GHS
3	Benzensulphonylchloride	98-09-9	1	20	28	1
10	Ethylhexyl acid phosphate ester	12,645-31-7	1	12	5	1
14	4-Tert-Butylcatechol (85%, probably in methanol)	98-29-3	1	9	8	1
17	Benzethonium chloride (10%)	121-54-0	1	6	5	1
34	Hydroxyethyl acrylate	818-61-1	1	38	54	2
68	Cetylpyridinium chloride (10%)	6004-24-6	1	7	5	1
69	Cetyltrimethyl ammonium bromide (CTAB) (10%) (1 of 2)	57-09-0	1	15	7	1
71	Di(2-ethylhexyl)sodium sulphosuccinate (10%)	577-11-7	1	4	4	1
77	Stearyltrimethylammonium chloride (10%)	112-03-8	1	13	9	1
129	N-Octylamine	111-86-4	1	3	4	1
^**^	1-Hexadecanaminium, N, N, N-trimethyl-, chloride (25%)	112-02-7	1	8	5	1
167	2-Ethyl-1-hexanol	104-76-7	2A	9	9	1
170	Cyclopentanol	96-41-3	2A	22	75	2
173	Gamma-Butyrolactone (1 of 2)	96-48-0	2A	46	192	2
176	Methyl acetate (1 of 2)	79-20-9	2A	46	115	2
180	N-Hexanol	111-27-3	2A	22	71	2
183	Propasol Solvent P	1569-01-3	2A	33	125	2
187	Lauryl sulphobetaine (10%)	14,933-08-5	2A	36	122	2
202	Furfural	98-01-1	2A	34	59	2
204	Methyl cyanoacetate	105-34-0	2A	80	68	0
209	N-Lauroyl sarcosine Na salt (10%)	137-16-6^*^	2A	14	105	2
218	2-Methyl-1-pentanol	105-30-6	2B	35	122	2
219	3-Chloropropionitrile	542-76-7	2B	19	59	2

^*^7631-98-3 corresponds to N-Dodecyl sarcosine Na salt or Sodium lauryl sarcosine (Gautheron et al. 1994)

^**^Test substance is not included in DRD but referred to DASF (Alépée et al. 2023)

Raw TEER-values were plotted as data bars for each time point from pre, post, 7–14 days (Figs. 5, 6). The data bars represent the average of TEER-measurements obtained from three RCE models per condition at each time point (Figs. 5, 6). Thus, the TEER-mean of each liquid and control per time point contains 9 TEER-values in Ohm*cm^2^. In total, three independent experimental runs were performed using three different biologic donors.

Beside the representation of raw TEER, the chemical classification is based on the membrane barrier functionality (%) of RCE triplicates treated with the same chemical. The barrier functionality (%) is calculated within each condition by normalizing post-exposure TEER to the respective TEER pre values. This means, a single model within a condition that is measured on day 0 post, 7 days and 14 days was normalized to its pre-TEER-value, allowing individual models to be accurately assessed over time (Table 3).

Discrimination of Cat. 1 and Cat. 2 liquids were shown by raw TEER-values (Ohm*cm^2^, Figs. 5, 6). To predict the classification of chemicals, tissue induced damages of Cat. 1 liquids were non-invasively measured over 14 days and compared with effects of Cat. 2 liquids (Fig. 5). 11 out of 11 Cat. 1 liquids demonstrated a significantly reduced TEER from pre to post (*p* < 0.0001, Fig. 5) in the RCE models. To induce irreversible damage by Cat. 1 liquids on the RCE models, TEER was not expected to increase over time. Indeed, no significant (ns) increase of TEER values from TEERpost to TEER14d was shown for all Cat. 1 liquids. RCE models treated with Hydroxyethyl acrylate, No. 34 indicated increased TEER from post to day 7 and 14 without significance (Fig. 5). However, except for chemical Hydroxyethyl acrylate, No. 34, 10 out of 11 Cat. 1 liquids induced significant damage on RCE models until day 14 after exposure. In summary, when TEERpost was compared to TEER14d values, no significant increase was observed in RCE models for all 11 chemicals.

Initial loss of TEER could also be observed in RCE models after exposure to Cat. 2 liquids (Fig. 6). A significant decrease in *TEER*_*1000Hz*_ from pre to post measurements was observed for 10 out of 12 Cat. 2 liquids with p values for 2-Ethyl-1-hexanol, No. 167 (*p* < 0.0001), Cyclopentanol, No. 170 (*p* < 0.0001), Methyl acetate, No. 176 (*p* = 0.0097), N-Hexanol, No. 180 (*p* < 0.0001), Propasol Solvent P, No. 183 (*p* = 0.0435), Lauryl sulphobetaine (10%), No. 187 (*p* = 0.0206), Furfural, No. 202 (*p* < 0.0001), N-Lauroyl sarcosine Na salt (10%), No. 209 (*p* = 0.0003), 2-Methyl-1-pentanol, No. 218 (*p* = 0.0003), 3-Chloropropionitrile, No. 219 (*p* < 0.0001) (Fig. 6). Two chemicals, Gamma-Butyrolacetone, No. 173 and Methyl cyanoacetate, No. 204 indicated less severe tissue damage by decreased TEERpre to TEERpost values: with 1597 ± 574.75 to 1179 ± 628.44 Ohm*cm^2^ for Gamma-Butyrolacetone (*p* = 0.4779) and values ranging from 1742 ± 405.67 to 1339 ± 340.9 Ohm*cm^2^ for Methyl cyanoacetate (*p* = 0.5101).

For GHS Cat. 2, 10 out of 12 liquids indicated increased TEER-mean of treated RCE models on day 7 and 14 after exposure when compared to TEERpost (Fig. 6). Significantly increased TEERpost to TEER14d values were measured for 7 Cat. 2 liquids: from 1179 ± 806,23 to 2093 ± 737,03 Ohm*cm^2^ for Gamma-Butyrolactone, No. 173 (*p* < 0,0001), from 509 ± 181.39 to 1384 ± 463.63 Ohm*cm^2^ for Methyl acetate, No. 176 (*p* = 0.0147), from 426 ± 131.41 to Ohm*cm^2^ for N-Hexanol, No. 180 (*p* = 0.0099), from 413 ± 61.49 to 1241 ± 652.56 Ohm*cm^2^ for Propasol Solvent P, No. 183 (*p* = 0.0238), from 612 ± 739.29 to 1558 ± 353.91 Ohm*cm^2^ for Lauryl sulphobetaine (10%), No. 187 (*p* = 0.0067), from 209 ± 79.31 to 1590 ± 1091.44 Ohm*cm^2^ for N-Lauroyl sarcosine Na salt (10%), No. 209 (*p* < 0.0001) and from 549 ± 209.36 to 1576 ± 947.74 Ohm*cm^2^ for 2-Methyl-1-pentanol, No. 218 (*p* = 0.0026). As exception, chemical 2-Ethyl-1-hexanol, No. 167 showed irreversible effects on tissue models indicated by low TEER-mean from post to 14 days.

The membrane barrier functionality (%) was evaluated by the normalized TEER-values to TEERpre of each condition (Table 3). As classified into GHS Cat. 1, 10 out of 11 Cat. 1 liquids significantly decreased membrane barrier functionality of RCE models below 30% after chemical exposure. For 6 out of 11 Cat. 1 liquids, TEERpost indicated below 10% of the barrier functionality when normalized to TEERpre of intact RCE models.

Compared to the UN GHS classification, ten out of 12 Cat. 2 liquids were correctly classified by the ImAi-test as Cat. 2 chemicals. In the case of tissue recovery, the mean of TEER14d was 104.5% ± 21.2. TEERpost values below 10% of the membrane barrier functionality showed no increase in TEER until day 14 which was observed for chemical 2-Ethyl-1-hexanol, No. 167 (Table 3). Chemical Methyl cyanoacetate, No. 204, indicated no tissue recovery with TEER values ranging from 80% ± 30 to 68% ± 41 at post and 14 days.

## Discussion

Human cell-based in vitro tissues are useful tools for assessing chemical hazards. A number of alternative approaches has been developed and officially adopted as OECD TGs as alternative to the in vivo Draize eye test. Current and future developments of NAMs can reduce in vivo application by aiming to fully replace the Draize eye test. For the endpoint skin sensitization, NAMs could already replace in vivo testing for consumer safety (OECD 2023a). For ocular safety, NAMs reach promising predictions of chemical hazards, but still indicate limitations for some chemical functional groups and problematic substances. The reliable distinction of reversible and irreversible ocular hazards across a broad range of chemicals remains a critical challenge for NAM development.

We aimed to develop the ImAi-test as standalone method to complement existing NAMs (OECD 2024a, 2024c) by integrating long-term assessment of in vitro tissue damage to distinguish reversible from irreversible effects. Considering the data presented herein, the ImAi-test promises to support the overall assessment of chemical hazard potential to the human eye by NAMs.

### Establishment of chemical reference list for eye-irritation testing

The collection of reference chemicals was an initial step toward the development of the ImAi-test method. The chemical selection was based on comprehensive research of different data bases containing in vivo data such as the published DRD (Barroso et al. 2017; ECETOC 1998. Eye Irritation Reference Chemicals Data Bank. Technical Report No.). To ensure high data quality, only recommended chemicals of the DRD were selected. Furthermore, chemicals had to be commercially available and represent different chemical classes, including the main drivers of classification. In addition, the chemical selection encompasses substances from all three UN GHS categories, including solids and liquids. This enables the identification of the applications and limitations of the developed test method.

### Development of an impedance spectroscopy measuring device

Impedance spectroscopy allows for the sensitive detection of membrane barrier properties and tissue integrity, including the assessment of time-dependent effects, through the measurement of electrical resistances in the tissues (Groeber et al. 2015). To analyze tissue damage in vitro, impedance spectroscopy is a useful method. During the development of the ImAi-test, the impedance spectroscopy CSM 2100 device was developed to identify and classify the hazard potential of chemicals on RCE models over time. Chemical-induced tissue damages of in vitro models were non-invasively monitored via TEER. For eye-irritation endpoint measurements, TEER-analysis is already integrated in the officially adopted OCED TG 494 (OECD 2023f). The guideline recommend low-voltage resistance devices with alternating current of 50–1000 Hz (OECD 2023f). During the development of the Vitrigel®-Eye Irritancy test method, a Millicell-ERS (Millipore) device (Takezawa et al. 2011) was used which is mostly set to 12.5-Hz measurements and is common for monolayer (Linz et al. 2020). The developed impedance spectrometer CSM 2100 was specifically adapted to 3D in vitro models to reach robust full spectrum measurements ranging from 1 to 200,000 Hz of each individual model over time. However, the TEER application of TG 494 differs from the ImAi-test method also in terms of measurement time points. Regarding non-invasive analysis of tissue recovery, the TG 494 only analyzes tissue damages for maximal 180 seco to identify chemicals not inducing eye irritation or corrosion and includes no repeated measurements over time as the in vivo test. (OECD 2023f, 2023b). The ImAi-test aimed to enable insights into tissue alterations until day 14, especially with focus on recovery after treatment with GHS Cat. 2 chemicals. The automatic CSM 2100 device provides standardized measurements compared to handheld devices e.g., chop-stick electrodes (Jovov et al. 1991). This enhances the method’s transferability for use in multiple laboratories. In addition to ocular chemical hazard identification, impedance spectroscopy has already been transferred to other human-relevant toxicological endpoints in vitro (Shaughnessey et al. 2022). This technique allows for a thorough analysis of different measurement parameters, including phase, amplitude, and impedance values over a frequency of 1 to 200,000 Hz. Full spectrum measurements can be particularly useful in predicting a wide range of in vitro eye irritations, including problematic substances such as colorant chemicals, detergents, surfactants and dilutions or formulations. By the incorporation of all measurement parameters, impedance spectroscopy could broaden the applicability domain of eye-irritation testing.

### Discrimination between category 1- and category 2-induced tissue damages following exposure to liquid substances

To improve the in vitro prediction of eye irritation caused by Cat. 2 chemicals, we have developed a test method to distinguish reversible from irreversible tissue damage of treated in vitro RCE models. Thus, we analyzed a selection of 23 liquids from the training set that induces severe eye damage and eye irritation (Cat. 1/Cat. 2). The discrimination of chemicals hazard potential relies on the recovery of the membrane barrier functionality of the 3D models after chemical exposure over time.

Following Draize eye test data, all tested Cat. 2 and Cat. 1 chemicals indicated irritation to the RCE models by initial loss of membrane barrier functionality (TEERpost) after chemical exposure (OECD 2023b). 100% of tested Cat. 1 chemicals and 83.3% Cat. 2 chemicals showed a significant decrease in TEERpost, clearly indicating tissue damage immediately after treatment when compared to intact tissue barrier via TEERpre. At the time after treatment, no distinction can be made between Cat. 1 and Cat. 2 chemicals. For this reason, we have integrated sensitive TEER measurements of the models on different days after treatment. With focus on Cat. 2 chemicals, day 7 and 14 were selected for repeated TEER-measurements. TEER analyzation of tissue recovery until day 14 enables a clear discrimination of Cat. 2 chemicals from Cat. 1. The prediction model was adapted from Lotz et al. to 14 days which makes prediction comparable to in vivo Draize data (Lotz et al. 2018; OECD 2023b). Currently, only the TG 492B is able to differentiate between severe from eye irritation and No Cat. chemicals in vitro in a single method. Besides the in vitro TG 492B, the combination of existing NAMs combining in vitro and ex vivo methods into DAs (OECD 2024a) is adopted to the OCED to classify all GHS categories including Cat. 2 chemicals (Alépée et al. 2019a, 2019b, 2023). Thus, the ImAi-test results were mainly compared to in vitro approaches such as the TG 492B and the DAs of TG 467.

Tested Cat. 1 liquids included 11 substances with various chemical classes, properties and main drivers of classification (CO = 4). The initial tissue damage was found to be significant in 100% of the tested liquids. To ensure the prediction of severe eye damage of the applied Cat. 1 liquids, the tissue properties were analyzed until day 14. The non-invasive monitoring of the tissue via TEER enabled the quantification of the membrane barrier functionality, which corresponds to the integrity of the tissues. Consequently, low electrical tissue resistance over time following the treatment of Cat. 1 liquids indicated irreversible damage with no tissue recovery. Based on the published prediction model, chemicals were classified into GHS categories after 14 days (Lotz et al. 2018). Thus, 90.9% of the tested Cat. 1 liquids (10/11) indicated irreversible damaging potential after exposure to the RCE models (Table 4).

**Table 4:** The ImAi-classification of the Category 1 liquids reached comparable results when compared to existing NAMs for the liquids

Correct ImAi-test classification of chemical (No. given by the DRD)	Correct classification by NAMs	References
Benzensulphonylchloride, No. 3	DA based on Short-Time-Exposure (STE) test method/BCOP	Alépée et al. (2019a)
	DA of Reconstructed human Corneal Epithelium (RhCE)/BCOP	Alépée et al. (2019b)
	Time-To-Toxicity (TTT) test method	Alépée et al. (2020)
	Isolated Chicken Eye (ICE) test method	Eskes et al. (2021)
	Eye irritation test for sub-categorization of liquids (EyeIRR-IS assay)	Cottrez et al. (2021)
Ethylhexyl acid phosphate ester, No. 10	DA of surfactants (DASF)	Alépée et al. (2023)
	DA of STE/BCOP and RhCE/BCOP	Alépée et al. (2019a), Alépée et al. (2019b)
	TTT test method	Alépée et al. (2020)
	EyeIRR-IS assay	Cottrez et al. (2021)
Benzethonium chloride (10%), No. 17	DASF	Alépée et al. (2023)
	TTT test method	Alépée et al. (2020)
	EyeIRR-IS assay	Cottrez et al. (2021)
Cetylpyridinium chloride (10%), No. 68	DASF	Alépée et al. (2023)
	TTT test method	Alépée et al. (2020)
	EyeIRR-IS assay	Cottrez et al. (2021)
Stearyltrimethylammonium chloride (10%), No. 71	DASF	Alépée et al. (2023)
	TTT test method	Alépée et al. (2020)
N-Octylamine, No. 129	DA of STE/BCOP DA of RhCE/BCOP	Alépée et al. (2019a), Alépée et al. (2019b)
	TTT test method	Alépée et al. (2020)
	EyeIRR-IS assay	Cottrez et al. (2021)
1-Hexadecanaminium, N, N, N-trimethyl-, chloride (25%, marked with **)	DASF	Alépée et al. (2023)
	TTT test method	Alépée et al. (2020)

Only chemical Hydroxyethyl acrylate, No. 34 showed increasing TEER-values slightly over 50% of the barrier functionality on day 14. In line with the ImAi-prediction, chemical Hydroxyethyl acrylate, No. 34 was also predicted as Cat. 2 within the development of the TTT test method and the STE (Alépée et al. 2020; Adriaens et al. 2018). Also, the DA based on STE and BCOP test method showed no clear classification of chemical Hydroxyethyl acrylate, No. 34 within the STE test method (Alépée et al. 2019a). In contrast to in vitro classification, the in vivo Draize data assume irreversible effects of chemical Hydroxyethyl acrylate, No. 34 on the corneal opacity (Barroso et al. 2017; OECD 2005; Alépée et al. 2019b; Cottrez et al. 2021). Interestingly, the DA for severe eye irritation/eye irritation, combining TG 492 and TG 437, correctly classified chemical No. 34 into GHS Cat. 1 (Alépée et al. 2019b). Using the MTT analysis, which is based on the reduction of the blue dye MTT (3-(4,5-dimethylthiazol-2-yl)−2,5-diphenyltetrazolium bromide the formazan salt, colored chemicals or MTT reducers can represent limitations due to reduction or interference of the vital dye and can lead to misclassification. However, impedance spectroscopy, which is based on non-invasive measurements of TEER can be applied after any chemical exposure and is not limited by coloring effects which was shown e.g., for chemical Ethylhexyl acid phosphate ester, No. 10 (Table 3). Thus, the chemical set includes two chemicals (No. 69 and 129) which could have reductive properties on the MTT solution. No clear GHS Cat. 1 discrimination for chemical Cetyltrimethyl ammonium bromide (CTAB) (10%), No. 69 was made during the development of the SkinEthic HCE Time-To-Toxicity test method while chemical N-Octylamine, No. 129 was correctly identified (Alépée et al. 2020).

All 12 Cat. 2 chemicals induced tissue damage after chemical exposure due to reduced TEERpost-values of RCE models. Recovery from damage by increasing TEER14d values was observed in RCE models treated with chemicals Cyclopentanol, No. 170, Gamma-Butyrolactone, No. 173, Methyl acetate, No. 176, N-Hexanol, No. 180, Propasol Solvent P, No.183, Lauryl sulphobetaine (10%), No. 187, Furfural, No. 202, N-Lauroyl sarcosine Na salt (10%), No. 209, 2-Methyl-1-pentanol, No. 218 and 3-Chloropropionitrile, No. 219 (Table 5).

**Table 5:** The ImAi test correctly classified 10 chemicals as GHS Category 2 and is comparable to the following NAMs

Correct ImAi-test classification of chemical (No. given by the DRD)	Correct classification by NAMs	References
2-Methyl-1-pentanol, No. 218	DA of STE/BCOP	Alépée et al. (2019a)
	TTT test method	Alépée et al. (2020)
	EyeIRR-IS assay	Cottrez et al. (2021)
Gamma-Butyrolactone, No. 173	TTT test method	Alépée et al. (2020)
	EyeIRR-IS assay	Alépée et al. (2020); Cottrez et al. (2021)
Methyl acetate, No. 176	EyeIRR-IS assay	Cottrez et al. (2021)
N-Hexanol, No. 180	DA of STE/BCOP DA of RhCE/BCOP	Alépée et al. (2020); Alépée et al. (2019a, 2019b)
	TTT test method	Alépée et al. (2020)
Propasol Solvent P, No. 183	TTT test method	Alépée et al. (2020)
Lauryl sulphobetaine (10%), No. 187	DASF	Alépée et al. (2023)
Furfural, No. 202	DA of STE/BCOP DA of RhCE/BCOP	Alépée et al. (2019a, 2019b)
3-Chloropropionitrile, No. 219	EyeIRR-IS assay	Cottrez et al. (2021)
N-Lauroyl sarcosine Na salt (10%), No. 209	–	–
Cyclopentanol, No. 170	–	–

Contrarily to ImAi-classification, chemical Cyclopentanol, No. 170 was mainly overpredicted as Cat. 1 within single or combined NAMs such as the EyeIRR-IS assay (Cottrez et al. 2021), tests during the development of the SkinEthik HCE TTT test method (Alépée et al. 2020) and tests for the DA of TG 467: in vitro STE and BCOP (Alépée et al. 2019a), as well as in vitro RhCE test method and BCOP (Alépée et al. 2019b). Interestingly chemical Methyl acetate, No. 176 is used as positive control in TG 492 and TG 492B but is marked as “should not be used” within the DRD (Barroso et al. 2017; OECD 2024b, 2024c). Overprediction for Methyl acetate, No. 176 as Cat. 1 were found within in tests during the development of the EpiOcular™ eye-irritation test (Kaluzhny et al. 2011) and in DA (STE/BCOP and RhCE/BCOP) (Alépée et al. 2019a, 2019b). However, treatments with both chemicals indicated significant tissue recovery by TEER-measurements from post to 14 days after EIT. Chemical Propasol Solvent P, No. 183 was differently classified in several in vitro approaches: DA of the OECD TG 467 (OECD 2024a), as well as the EyeIRR-IS assay (Cottrez et al. 2021) have shown over-prediction of chemical Propasol Solvent P, No. 183. However, the ImAi-test as well as TG 492B were in line with the in vivo data of the DRD (ECETOC 1998. Eye Irritation Reference Chemicals Data Bank. Technical Report No.). The ImAi-classification of chemical Furfural, No. 202, that was in line with DA findings using in vitro RhCE and BCOP test methods (Alépée et al. 2019b), indicated tissue recovery over time in contrast to TG 492B which resulted in a classification as Cat. 1. Further the EyeIRR-IS assay overpredicted chemical Furfural, No. 202 as Cat. 1 (Cottrez et al. 2021). The ImAi-test could clearly measure increased membrane barrier functionality on day 7 and 14 after treatment with chemicals 2-Methyl-1-pentanol, No. 218 and 3-Chloropropionitrile, No. 219. In contrast, the GHS classification as Cat. 2 was not comparable with results of the DAL-1 (TG 467) for chemical 2-Methyl-1-pentanol, No. 218 (Alépée et al. 2019b) when tested on SkinEthic HCE tissue models. As well for chemical 3-Chloropropionitrile, No. 219, no clear Cat. 2 predictions could be made based on TG 467 and TG 492B (Alépée et al. 2019b, 2022).

To broaden the applicability domain, also problematic test substances (surfactants) were included in the ImAi-test. Chemical N-Lauroyl sarcosine salt (10%), No. 209 was correctly classified by ImAi-test as Cat. 2 chemical due to tissue recovery until day 14 after damage. Based on the DRD, N-Lauroyl sarcosine Na salt (10%), No. 209 should not be used as a test chemical (OECD 2023b). Due to the limited amount of available reference data to compare in vitro results, some Cat. 1 and Cat. 2 surfactants such as No. 209 were included in the study. Interestingly, the DA for surfactants based on SkinEthik™ HCE EIT and the modified STE protocol identified N-Lauroyl sarcosine salt (10%), No. 209 as Cat. 1 chemical (Alépée et al. 2023). Moreover, it was not possible to make a prediction for chemical No. 209 within the ex vivo ICE histopathology test (Eskes et al. 2021). In total, two Cat. 2 chemicals were misclassified within the test set: chemical 2-Ethyl-1-hexanol, No. 167 was overclassified into GHS Cat. 1 and chemical Methyl cyanoacetate, No. 204 was not identified as Cat. 2 as it showed only low decrease of TEER values after the application. Based on the DRD, chemical 2-Ethyl-1-hexanol, No. 167 is classified as a Cat. 2A chemical. Tissue damages by 2-Ethyl-1-hexanol, No. 167 require recovery of at least 21 days for proper classification. However, the initial tissue irritation indicated by low membrane barrier functionality after exposure to 2-Ethyl-1-hexanol, No. 167 showed no recovery until day 14 within the ImAi-test. The same overprediction of test substance 2-Ethyl-1-hexanol, No. 167 was found in the pre-validation study for TG 492 (Alépée et al. 2013). In contrast, No. 167 was correctly classified based on TG 467 and TG 492B (Alépée et al. 2019b, 2022). Interestingly, to enhance the accuracy of Cat. Two chemical classifications, OptiSafe Eye Irritation Test™ method proposes adding antioxidants to the tear film during non-animal testing (Lebrun et al. 2021b). The acellular *in chemico* approach has demonstrated a reduction in false positive chemical predictions and could prove useful in optimizing in vitro classification (Lebrun et al. 2021b). For chemical Methyl cyanoacetate, No. 204, no initial chemical injury in RCE models was observed via TEER in contrast to TG 492B and the EyeIRR-IS assay (Alépée et al. 2020; Cottrez et al. 2021; OECD 2024c). Interestingly, further NAM-based classification of chemical Methyl cyanoacetate, No. 204 showed similar tendencies to underpredict into GHS No Cat. in the DA of STE and BCOP, as well as the DA of RhCE using EpiOcular and BCOP (Alépée et al. 2019a, 2019b). Further, No. 204 was identified as false negative in the ICE histopathology test (Lebrun et al. 2021a). TEER-analysis indicated loss of tissue integrity after chemical treatment of chemical Methyl cyanoacetate, No. 204, however still too less to reach proper classification. Overall, the ImAi-test method has clear advantages in providing quantitative data on membrane barrier properties including tissue damage and regeneration over time.

In summary, the ImAi classification of 90.9% (*N* = 11) correctly identified Cat. 1 liquids was higher than the TG 492B with 85.4% of identified Cat. 1 liquids (*N* = 21) (OECD 2024c). Further, the ImAi-prediction of 83.3% (*N* = 12) of Cat. 2 liquids were also better compare to 79.8% (*N* = 25) of liquids tested in the TG 492B (OECD 2024c).

## Conclusion

The detection of a wide range of chemically induced eye damages with NAMs, in particular, the identification of Cat. 2 chemicals remain challenging. Furthermore, available DAs like TG 467 are time-consuming and cost intensive. Thus, further developments or improvements of existing NAMs are needed. The integration of impedance spectroscopy enables the quantification of post-exposure tissue effects up to 14 days. This allows the analysis of tissue regeneration, which is particularly important for the detection of Cat. Two chemicals, to be carried out on the in vitro model. The integration of impedance spectroscopy for the prediction of eye irritation has also been tested for diluted Cat. One chemical for a more precise labeling of the eye-irritation potential of products containing chemicals with eye-irritation potential (Weissinger et al. 2024). A long-term and non-destructive TEER-based analyzation of the treated RCE models would improve the prediction of in vitro ocular irritation.

In conclusion, the development of the human based ImAi-test aimed to complement existing NAMs with sensitive membrane barrier measurements by complementing the overall prediction of the eye-irritation potential of chemicals in non-animal approaches.

## Data availability statement

The data generated and/or analyzed in this study are available from the corresponding author upon reasonable request.

## Supplementary Information

Below is the link to the electronic supplementary material.

Supplementary file1 (DOCX 311 kb)

## Data Availability

The data generated and/or analyzed in this study are available from the corresponding author upon reasonable request.
