# Supplementary material for: Non-invasive in vitro NAM for the detection of reversible and irreversible eye damage after chemical exposure for GHS classification purposes (ImAi)
Source: Arch Toxicol. 2025 Jan 8;99(3):1011–28. doi: 10.1007/s00204-024-03940-x (PMC11821759; doi:10.1007/s00204-024-03940-x)
Supplement: Supplementary file 1 — Supplementary file1 (DOCX 311 kb) [file 204_2024_3940_MOESM1_ESM.docx]

1. **Supplementary material**

# Reference list

The established reference list includes detailed information about selected chemicals (Tab. S1). Chemicals of the validation set are marked with *.

Tab. S1: Set of 329 test chemicals including training and test. 30 test chemicals, consisting of 5 liquids and solids from each UN-GHS category, were selected for validation testing (marked with *). Colored and MTT-reducing chemicals are marked with ^C^.

More descriptions of corneal opacity, CO = 0: CO scores equal to 0 in all animals and all observed time points; CO > 0: in at least one observation time in at least one animal and all animals showing mean scores of days 1–3 below the classification cut-offs for all endpoints. ** indicates at least one animal with a mean score of days 1–3 above the classification cut-off for at least one endpoint. MTT interfering substances such as Methylene blue solution; 6-nitro-1,2,3,4-tetrahydroquinoxaline; 2-[(4-amino-2-nitrophenyl)amino]benzoic acid; N-allyl-N-(4-amino-2-nitrophenyl)amine; Basazol C Blue pr 8056 or lead to MTT reduction substances such as 3-Phenoxybenzyl alcohol, Ethyl thioglycolate, Piperonyl butoxide, p-Methyl thiobenzaldehyde, [3-(2-Aminoethylamino)propyl]trimethoxysilane, Methyl thioglycolate, Tetraethylene glycol diacrylate can be tested within the ImAi-test.

# Eye irritation test

The Eye irritation test (EIT) based on the OCED TG 492 was adapted for the ImAi-test to identify the hazard potential of liquids (Fig. S1).

##
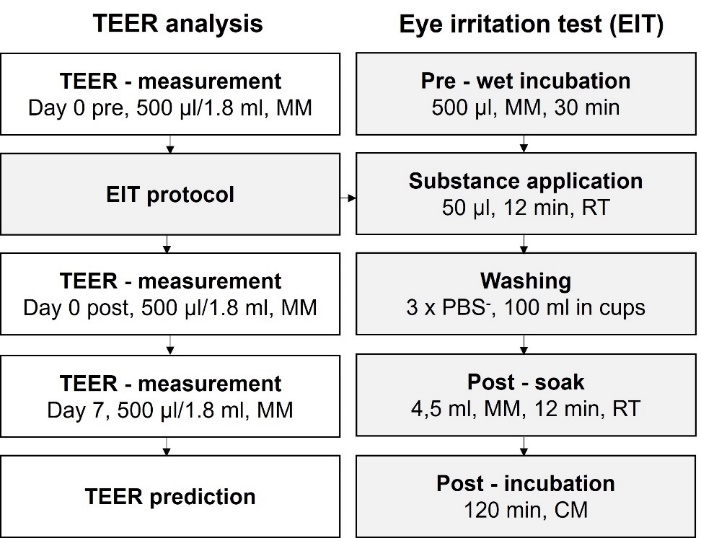


## Fig. S1: Protocol steps to perform an eye irritation test for liquids. MM indicates measuring media (2.3 impedance spectroscopy); CM indicates culture media (2.2 Tissue engineered RCE models)

# TEER data

TEER-measurements of negative control (distilled water) and positive control (Benzalkonium chloride 10%) before and after the application and from day 7 and 14. Raw and normalized values of both control groups were measured within the TEER quality range (Fig. S2).

##
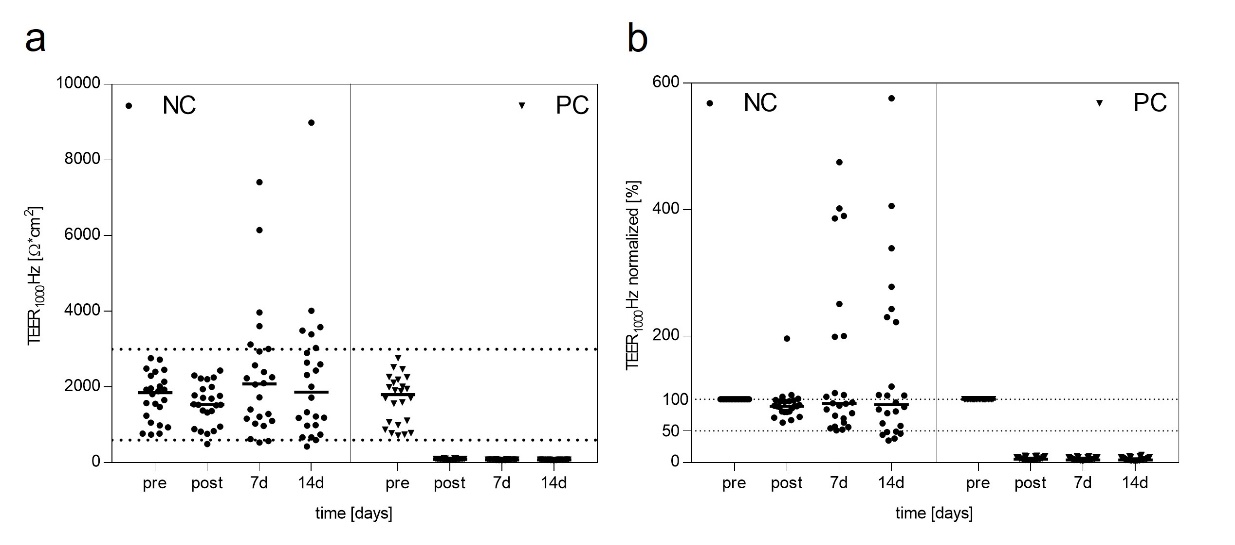


## Fig. S2: The TEER-values of the negative and positive controls are presented as raw data. a. The quality range for TEER values (Ohm*cm^2^) before the test (pre) is indicated by dotted lines and falls between 600 and 3000 Ohm*cm^2^. B. TEER values were normalized to day 0 prior to testing. Only models within the quality range were used for testing. TEER measurements were performed in triplicate across eight individual runs using three different biological donors. Each data point represents one model at the respective measurement time with deviation from the median.
